# *Phaeoisaria bailongtanensis*, *P. magnoliicola* and *P. qujingensis* spp. nov. (Pleurotheciaceae, Pleurotheciales) from Yunnan, China, with an augmented description of *Phaeoisaria clematidis*

**DOI:** 10.3897/mycokeys.137.199957

**Published:** 2026-07-21

**Authors:** Qiu-Ju Shang, Jing Yang, Kevin D. Hyde, Nalin N. Wijayawardene, Hind A. AL-Shwaiman, Abdallah M. Elgorban, Faten Zubair Filimban, Wannapawn Watsuntorn, Dong-Qin Dai

**Affiliations:** 1 Center for Yunnan Plateau Biological Resources, Protection and Utilization & Yunnan International Joint Laboratory of Fungal Sustainable Utilization in South and Southeast Asia, College of Biology and Food Engineering, Qujing Normal University, Qujing, Yunnan 655011, China Centre of Excellence in Fungal Research, Mae Fah Luang University Chiang Rai Thailand; 2 Key Laboratory of Yunnan Provincial Department of Education of the Deep-Time Evolution on Biodiversity from the Origin of the Pearl River, Qujing Normal University, Qujing, Yunnan Province, 655011, China High-Value Food from Mushrooms and Bioactive Plants in the Green Economy Value Chain Research Group, Institute of Biotechnology and Genetic Engineering, Chulalongkorn University Bangkok Thailand; 3 Langfang Normal University, Langfang 065000, Hebei, China Center for Yunnan Plateau Biological Resources, Protection and Utilization & Yunnan International Joint Laboratory of Fungal Sustainable Utilization in South and Southeast Asia, College of Biology and Food Engineering, Qujing Normal University Qujing China; 4 Centre of Excellence in Fungal Research, Mae Fah Luang University, Chiang Rai 57100, Thailand Key Laboratory of Yunnan Provincial Department of Education of the Deep-Time Evolution on Biodiversity from the Origin of the Pearl River, Qujing Normal University Qujing China; 5 CAS Key Laboratory for Plant Diversity and Biogeography of East Asia, Kunming Institute of Botany, Chinese Academy of Sciences, Kunming 650201, China CAS Key Laboratory for Plant Diversity and Biogeography of East Asia, Kunming Institute of Botany, Chinese Academy of Sciences Kunming China; 6 High-Value Food from Mushrooms and Bioactive Plants in the Green Economy Value Chain Research Group, Institute of Biotechnology and Genetic Engineering, Chulalongkorn University, 254 Phayathai Road, Pathumwan, Bangkok, 10330, Thailand Department of Botany and Microbiology, College of Science, King Saud University Riyadh Saudi Arabia; 7 Department of Bioprocess Technology, Faculty of Technology, Rajarata University of Sri Lanka, Mihintale 50300, Sri Lanka Center of Excellence in Biotechnology Research (CEBR), DSR, King Saud University Riyadh Saudi Arabia; 8 Department of Botany and Microbiology, College of Science, King Saud University, Riyadh 11451, Saudi Arabia Division of Plant Sciences, Department of Biological Sciences, Faculty of Sciences, King Abdulaziz University Jeddah Saudi Arabia; 9 Center of Excellence in Biotechnology Research (CEBR), DSR, King Saud University, Riyadh, Saudi Arabia Langfang Normal University Langfang China; 10 Division of Plant Sciences, Department of Biological Sciences, Faculty of Sciences, King Abdulaziz University, Jeddah, Saudi Arabia Department of Bioprocess Technology, Faculty of Technology, Rajarata University of Sri Lanka Mihintale Sri Lanka

**Keywords:** 3 new taxa, phylogeny, Sordariomycetes, taxonomy

## Abstract

Four phaeoisaria-like taxa were collected from decaying wood in Yunnan, China. Morphological examinations and phylogenetic analyses based on combined ITS, LSU, SSU, and *rpb2* sequence data strongly support the recognition of the three new species, *Phaeoisaria bailongtanensis*, *P. magnoliicola* and *P. qujingensis* within *Phaeoisaria* s.str. and confirm the occurrence of *P. clematidis* in Yunnan Province. Following re-examination of the original collections of *P. clematidis* from Germany, a lectotype and an isolectotype are designated for this species, and our fresh collection is designated as a reference specimen. Detailed comparisons with morphologically similar and phylogenetically related species are provided to highlight the distinctiveness of the new taxa. In addition, an updated phylogenetic tree of *Phaeoisaria* is presented to further clarify interspecific relationships within the genus.

## Introduction

*Phaeoisaria* (Pleurotheciaceae, Pleurotheciales, Sordariomycetes, fide Hyde et al. 2024) was established by Höhn. (1909) with *P. bambusae* as the type species. Asexual morphs of the genus exhibit erect, parallel-arranged synnemata (when present), polyblastic, sympodial, proliferating conidiogenous cells with prominent denticulations, and hyaline to pale brown, aseptate or septate, ellipsoidal, obovoid to fusiform conidia (de Hoog and Papendorf 1976; Réblová et al. 2016). Sexual morphs of the genus *Phaeoisaria* have been reported only for *Phaeoisaria filiformis*, which is characterised by black, perithecial ascomata with a two-layered peridium, filamentous, branched, septate paraphyses, unitunicate, 8-spored, cylindrical-clavate asci, and multi-septate, hyaline, filiform ascospores tapering at both ends (Luo et al. 2019). Forty-four epithets are listed under *Phaeoisaria* in Index Fungorum (https://indexfungorum.org/Names/Names.asp, accessed 15 May 2026). Of these, approximately 30 species are currently accepted, of which 25 have sequence data (Cheng et al. 2014; Luo et al. 2018, 2019; Liu et al. 2022; Xu et al. 2024; Li et al. 2025; Luo et al. 2025; Réblová et al. 2025; Zhang et al. 2025; Tian et al. 2026).

Geographically, *Phaeoisaria* species are widely distributed, with records from Africa, the Americas, Asia, Australia, Europe, and Oceania (Liu et al. 2022; Réblová et al. 2025). Ecologically, members of *Phaeoisaria* are predominantly saprobes with remarkable adaptability, occurring on diverse substrates in freshwater (e.g., submerged wood), marine (e.g., intertidal sediment) and terrestrial (e.g., soil and plant debris) habitats (Cheng et al. 2014; Crous et al. 2015; Luo et al. 2018; Hyde et al. 2019; Liu et al. 2022; Luo et al. 2025; Réblová et al. 2025; Wang et al. 2025; Zhang et al. 2025; Tian et al. 2026). They play critical roles in ecosystem functioning by driving lignocellulose decomposition and nutrient cycling (Hyde et al. 2016; Liu et al. 2022; Li et al. 2024). Additionally, some *Phaeoisaria* species display opportunistic pathogenicity in humans; for instance, *P. clematidis* and an unidentified *Phaeoisaria* species have been reported as causal agents of keratitis in immunocompromised individuals (Guarro et al. 2000; Zhao et al. 2025).

Current research on *Phaeoisaria* has advanced from morphology-based classification to integrative taxonomy, relying on multi-locus phylogenetic analyses (based on ITS, LSU, SSU, *rpb2* and *tef1α* loci) to resolve species boundaries and uncover cryptic diversity (Réblová et al. 2016, 2025; Wang et al. 2025; Zhang et al. 2025). However, significant knowledge gaps remain. Some previously described species lack molecular data, sexual morphs are poorly documented, and the functional diversity and biogeographical drivers of the genus are still incompletely understood (Réblová et al. 2016, 2025; Wang et al. 2025).

During a survey of hyphomycetous fungi on decaying wood in southwestern China, four *Phaeoisaria* taxa were collected from Yunnan Province. Based on morphological and phylogenetic evidence, three species, *P. bailongtanensis*, *P. magnoliicola* and *P. qujingensis* are introduced as new species, and the known species *P. clematidis* is reported. Moreover, after re-examining the original syntype collections of Fuckel (1870) and our fresh collections, we designate a lectotype, an isolectotype and a reference specimen for *P. clematidis*. Descriptions and illustrations are provided for these four species, along with an updated phylogenetic tree for *Phaeoisaria*.

## Materials and methods

### Herbarium specimens and specimen collection

The syntype specimens of *Phaeoisaria clematidis* were loaned from the herbaria of the Conservatoire et Jardin botaniques de la Ville de Genève (G), Switzerland. Fresh samples on dead wood were collected from different localities in Yunnan Province, China. The specimens were transported to the laboratory in plastic zip-lock bags and maintained at room temperature.

### Morphological observations

The microscopic characteristics of *P. clematidis* were examined as follows. Small fragments (3–5 mm) bearing synnemata were removed from the main specimens and rehydrated with water or 5% KOH for 3–5 minutes prior to examination. Specimens were examined following the methods detailed in Senanayake et al. (2020). Macroscopic observations and photography were performed using a Motic SMZ-140 dissecting microscope and a Discovery V8 stereo microscope for herbarium specimens, and a LEICA S8 APO optical microscope (Leica Microsystems, Wetzlar, Germany) for fresh specimens. Microscopic examination and photography were conducted using a Nikon Eclipse 80i compound microscope equipped with a Canon EOS 600D camera for herbarium specimens, and an Olympus BX53 compound microscope (Olympus, Tokyo, Japan) fitted with an Olympus DP74-CU camera for fresh specimens. Measurements were made using the Tarosoft(R) Image Frame Work software (Version 0.9.7) (Liu et al. 2010). Images were processed using Adobe Photoshop CS6 Extended v. 13.0 software. The newly collected specimens were deposited in the Herbarium of Cryptogams of Kunming Institute of Botany, Chinese Academy of Sciences (HKAS), Kunming, China, with duplicates in the Herbarium of Guizhou Medical University (GMB-W), Guiyang, China. Living cultures were obtained by single spore isolation as described in Senanayake et al. (2020). The cultures were grown on potato dextrose agar (PDA) and deposited in the Guizhou Medical University Culture Collection (GMBCC), Guiyang, China.

### Taxonomic nomenclature and registration

Type designations follow the International Code of Nomenclature for algae, fungi, and plants (ICN). Facesoffungi numbers and Index Fungorum registration identifiers for the new species and newly typified taxa were obtained according to Jayasiri et al. (2015) and Index Fungorum (2026), respectively. New taxa were established following the recommendations outlined by Jeewon and Hyde (2016) and Aime et al. (2021).

### DNA extraction, PCR amplification and sequencing

Total genomic DNA was extracted from fresh fungal mycelium (scraped from colony margins of cultures grown on PDA at 25–30 °C for 1–4 weeks) using the Biospin Fungus Genomic DNA Extraction Kit (BioFlux®, China), following the manufacturer’s instructions.

The DNA amplification was performed by polymerase chain reaction (PCR) as described below. The internal transcribed spacer (ITS) region, large subunit rDNA (LSU), small subunit rDNA (SSU) and RNA polymerase II second largest subunit (*rpb2*) regions were amplified using the primer pairs ITS4/ITS5 (White et al. 1990), LR0R/LR5 (Vilgalys and Hester 1990), NS1/NS4 (White et al. 1990) and fRPB2-5F/fRPB2-7cR (Liu et al. 1999), respectively. A total of 25 µL PCR reaction mixture was prepared, containing 2.0 µL template DNA, 1.0 µL forward primer, 1.0 µL reverse primer, 12.5 µL 2× Master Mix and 8.5 µL ddH_2_O. The PCR thermal cycling conditions for the ITS, LSU and SSU regions were initially at 94 °C for 3 min, followed by 35 cycles of denaturation at 94 °C for 30 s, annealing at 55 °C for 50 s, elongation at 72 °C for 1 min and a final extension at 72 °C for 10 min. For the *rpb2* locus, PCR conditions were initially at 95 °C for 5 min, followed by 35 cycles of denaturation at 95 °C for 1 min, annealing at 52 °C for 2 min, elongation at 72 °C for 90 s, and a final extension at 72 °C for 10 min. Purification and sequencing of the PCR products, using the same primers, were performed by Shanghai Sangon Biological Engineering Technology and Services Co., Ltd. (Shanghai, China).

### Phylogenetic analyses

The sequences generated in this study were analyzed together with reference sequences of related taxa retrieved from GenBank (Table 1) and recent publications (Li et al. 2025; Luo et al. 2025; Réblová et al. 2025; Wang et al. 2025; Zhang et al. 2025; Tian et al. 2026), including sequences of *Phaeoisaria* species and other representative genera within Pleurotheciaceae. The consensus sequences were initially aligned using MAFFT v. 7.310 (Katoh and Standley 2013) and further adjusted manually where necessary in Bioedit v. 7.0.9.1 (Hall 1999). Individual gene datasets were initially analyzed separately, and the recovered phylogenies were compared. The partition homogeneity test (PHT) was performed with PAUP v. 4.0b10 (Swofford 2002) to determine whether datasets were congruent and could be combined. The four gene regions were concatenated into a combined dataset using SequenceMatrix v. 1.7.8. The best-fit evolutionary model for each gene partition was selected using MrMTgui v.1.0 (Nylander 2004; Nuin 2007). Phylogenetic trees were generated from maximum likelihood (ML) and Bayesian inference (BI) analyses, which were performed following the methods of Dissanayake et al. (2020). Preliminary ML trees were generated via the CIPRES Science Gateway, and the final ML tree (RAxML v. 1.5b2; Silvestro and Michalak 2012) was constructed using raxmlGUI with 1000 rapid bootstrap replicates under the GTR+GAMMA+I substitution model.

**Table 1.** Taxa used in Pleurotheciaceae and their GenBank accession numbers.

**Taxa**	**Strain Numbers**	**GenBank Accession Numbers**				**References**
		**ITS**	**LSU**	**SSU**	***rpb2***	
*Phaeoisaria annesophieae*	CBS 143235^T^	MG022180	MG022159	–	–	Crous et al. (2017)
*Phaeoisaria annesophieae*	MFLUCC 19-0325	MT559109	MT559084	–	–	Shi et al. (2021)
*Phaeoisaria aquatica*	MFLUCC 16-1298^T^	MF399237	MF399254	–	MF401406	Luo et al. (2018)
*Phaeoisaria aquatica*	MFLUCC 23-0051	OQ913674	OQ913689	OQ913710	–	Luo et al. (2018)
***Phaeoisaria bailongtanensis***	**GMBCC 2513^T^**	**PZ556860**	**PZ428987**	**PZ426421**	**PZ580738**	**This study**
***Phaeoisaria bailongtanensis***	**GMBCC 2514**	**PZ556861**	**PZ428988**	**PZ426422**	**PZ580739**	**This study**
*Phaeoisaria clematidis*	MFLUCC 16-1273	MF399229	MF399246	–	–	Luo et al. (2018)
*Phaeoisaria clematidis*	MFLUCC 17-1341	MF399230	MF399247	MF399216	MF401400	Luo et al. (2018)
*Phaeoisaria clematidis*	DAOM 226789	JQ429155	JQ429231	JQ429243	JQ429262	Réblová et al. (2012)
***Phaeoisaria clematidis***	**GMBCC 2517**	**PZ556867**	**PZ428989**	**PZ426423**	**PZ580744**	**This study**
***Phaeoisaria clematidis***	**GMBCC 2518**	**PZ556868**	**PZ428990**	**PZ426424**	**PZ580745**	**This study**
***Phaeoisaria clematidis***	**GMBCC 2522**	**PZ556862**	**–**	**–**	**–**	**This study**
*Phaeoisaria dalbergiae*	CPC 39540^T^	OK664703	OK663742	OK663796	OK651159	Crous et al. (2021)
*Phaeoisaria diversa*	ZG0001^T^	MG237857	MG237859	–	–	Luo et al. (2025)
*Phaeoisaria diversa*	ZG0023	MG237858	MG237860	–	–	Luo et al. (2025)
*Phaeoisaria ellipsoidea*	IFRDCC 3134^T^	ON533383	ON533387	–	–	Yang et al. (2023)
*Phaeoisaria ellipsoidea*	BAB-4787	KR154997	–	–	–	Yang et al. (2023)
*Phaeoisaria fasciculata*	CBS 127885^T^	KT278719	KT278705	KT278693	KT278741	Réblová et al. (2016)
*Phaeoisaria fasciculata*	DAOM 230055	KT278720	KT278706	KT278694	KT278742	Réblová et al. (2016)
*Phaeoisaria filiformis*	MFLUCC 18-0214^T^	MK878381	MK835852	MK834785	–	Luo et al. (2019)
*Phaeoisaria goiasensis*	FCCUFG 02^T^	MT210320	MT375865	–	–	Jayawardena et al. (2022)
*Phaeoisaria goiasensis*	FCCUFG 03	MT210321	MT375866	–	–	Jayawardena et al. (2022)
*Phaeoisaria guiyangensis*	GZCC 25-0626^T^	PV871233	–	PV871241	PV872884	Zhang et al. (2025)
*Phaeoisaria guiyangensis*	GZCC 25-0627	PV871234	–	PV871242	PV872885	Zhang et al. (2025)
*Phaeoisaria guttulata*	MFLUCC 17-1965^T^	MG837021	MG837016	MG837026	–	Hyde et al. (2018)
*Phaeoisaria guttulata*	KUNCC 23-15646	PQ644532	PQ650101	PQ844518	–	Hyde et al. (2018)
*Phaeoisaria laianensis*	CCTCC AF 2022069^T^	ON937559	ON937557	ON937562	–	Liu et al. (2022)
*Phaeoisaria laianensis*	CCTCC AF 2022073	ON937560	ON937561	ON937558	–	Liu et al. (2022)
*Phaeoisaria loranthacearum*	BYCDW25	MG820097	–	–	–	Xu et al. (2020)
*Phaeoisaria loranthacearum*	BYCDW24	MG820098	–	–	–	Xu et al. (2020)
*Phaeoisaria loranthacearum*	CBS 140009^T^	KR611888	MH878676	–	–	Crous et al. (2015)
*Phaeoisaria loranthacearum*	KUNCC 24-18588	PV264829	PV264838	PV335230	–	Wang et al. (2025)
***Phaeoisaria magnoliicola***	**GMBCC 2523^T^**	**PZ556863**	**PZ428991**	**PZ426425**	**PZ580740**	**This study**
***Phaeoisaria magnoliicola***	**GMBCC 2524**	**PZ556864**	**PZ428992**	**PZ426426**	**PZ580741**	**This study**
*Phaeoisaria microspora*	MFLUCC 16-0033^T^	MF671987	MF167351	–	MF167352	Hyde et al. (2017)
*Phaeoisaria mononematosa*	CGMCC 3.28757^T^	PV264830	PV264839	PV335231	PV298272	Wang et al. (2025)
*Phaeoisaria motuoensis*	KUNCC 10410^T^	OP626333	OQ947034	OQ947036	–	Xu et al. (2024)
*Phaeoisaria obovata*	KUNCC 23-15598	PP049489	PP049505	PP049523	PP068784	Wang et al. (2024)
*Phaeoisaria obovata*	CGMCC 3.27015^T^	PP049488	PP049504	PP049522	PP068788	Wang et al. (2024)
*Phaeoisaria parallela*	CBS 153403^T^	PV455936	PV455950	PV455963	PV483450	Réblová et al. (2025)
*Phaeoisaria pseudoclematidis*	MFLUCC 11-0393^T^	KP744457	KP744501	KP753962	–	Liu et al. (2015)
***Phaeoisaria qujingensis***	**GMBCC 2515^T^**	**PZ556865**	**PZ428993**	**PZ426427**	**PZ580742**	**This study**
***Phaeoisaria qujingensis***	**GMBCC 2516**	**PZ556866**	**PZ428994**	**PZ426428**	**PZ580743**	**This study**
*Phaeoisaria sedimenticola*	CGMCC 3.14949^T^	JQ074237	JQ031561	–	–	Cheng et al. (2014)
*Phaeoisaria sedimenticola*	S-908	MK878380	MK835851	–	–	Luo et al. (2019)
*Phaeoisaria siamensis*	MFLUCC 16-0607^T^	MK607610	MK607613	MK607612	MK607611	Hyde et al. (2019)
*Phaeoisaria sparsa*	FMR 11939	PV455937	PV455951	PV455964	PV483451	Réblová et al. (2025)
*Phaeoisaria synnematica*	NFCCI 4479^T^	MK391494	MK391492	–	–	Boonmee et al. (2021)
*Phaeoisaria xishuangbannaensis*	GZCC 25-0676^T^	OR762191	OR762297	OR763354	–	Tian et al. (2026)
*Phaeoisaria yadongensis*	KUNCC 24-17782^T^	PQ404918	PQ404920	PQ404922	–	Li et al. (2025)
*Phaeoisaria yadongensis*	KUNCC 24-17783	PQ404919	PQ404921	PQ404923	–	Li et al. (2025)
*Pleurotheciella rivularia*	CBS 125237	JQ429161	JQ429233	JQ429245	JQ429264	Luo et al. (2018)
*Pleurotheciella rivularia*	CBS 125238^T^	JQ429160	JQ429232	JQ429244	JQ429263	Luo et al. (2018)

Superscript T denotes ex-type strains. The en-dash (–) indicates that the corresponding gene sequence of this species is unavailable in GenBank. The newly generated sequences are indicated in bold. Additional *tef1α* sequences generated in this study are provided in the taxonomic treatments but were not included in the phylogenetic analyses.

The BI analysis was performed using MrBayes v. 3.2.7a on ACCESS via the CIPRES Science Gateway. The dataset was partitioned by gene (ITS, LSU, SSU, *rpb2*), with substitution models set as follows: ITS, LSU, SSU and *rpb2*: nst = 6 with ingamma-distributed rates. MCMC runs were conducted with 6 chains for 10,000,000 generations, and the first 25% of trees were discarded as burn-in.

To evaluate potential recombination between the new species and phylogenetically allied taxa, pairwise homoplasy index (PHI) tests were performed using SplitsTree based on the concatenated multi-locus dataset comprising ITS, LSU, SSU and *rpb2* sequences. The interleaved NEXUS and PHYLIP files for BI, PHI and ML analyses were formatted with the ALTER online program (Glez-Pena et al. 2010).

Phylograms were plotted in FigTree v. 1.4.0 (Rambaut 2012) and edited in Adobe Illustrator CS5 v. 15.0.0. (Adobe Systems, USA). Sequences derived from this study were deposited in GenBank (Table 1). The final alignment and phylogenetic tree were submitted to TreeBASE under submission ID 32690 (TreeBASE 2026).

## Results

### Phylogenetic analysis

**Figure 1.**
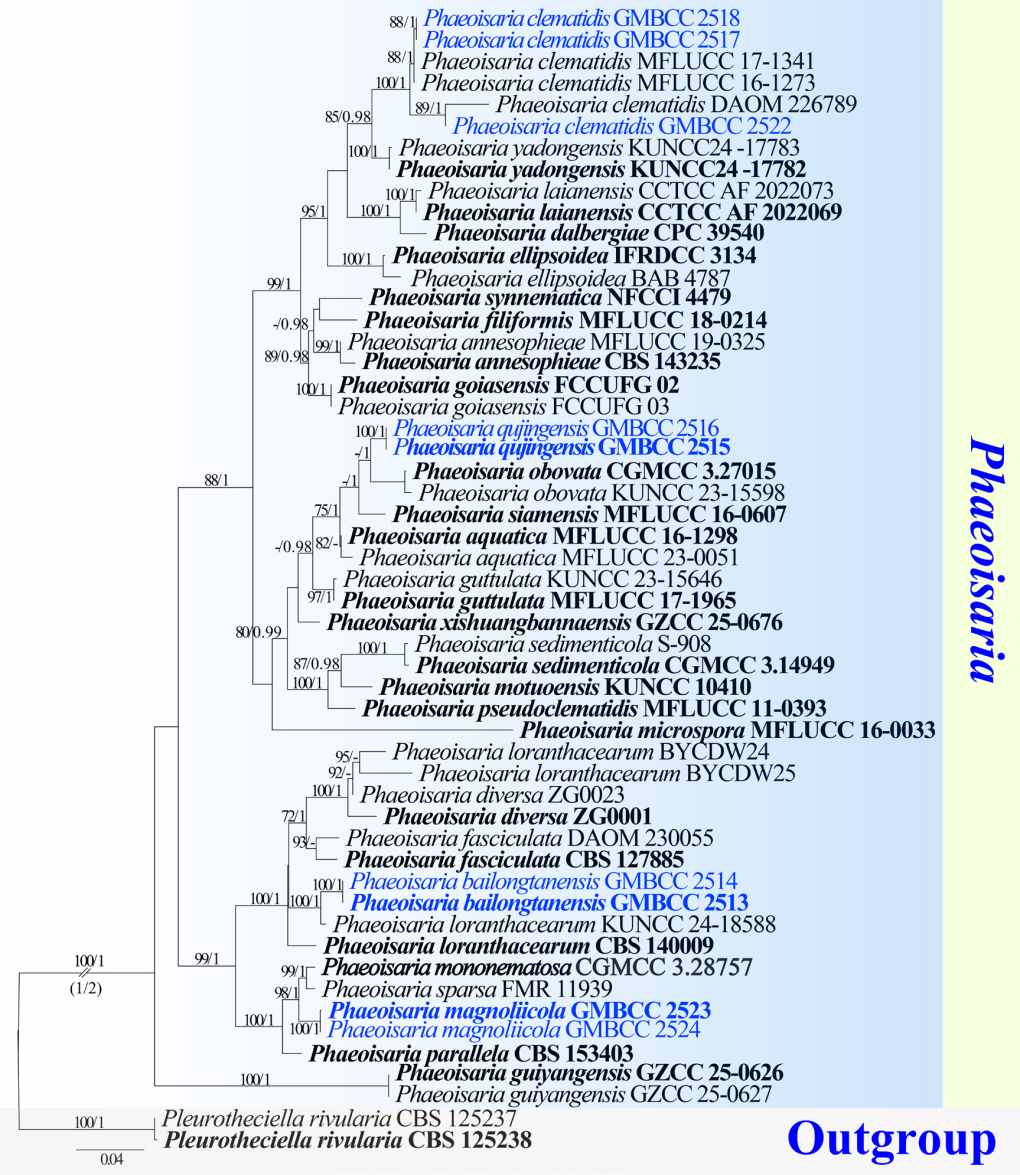
Phylogram generated from ML analyses for *Phaeoisaria* using ITS, LSU, SSU and *rpb2* sequence data. Bootstrap support values for ML equal to or greater than 75% and BI values greater than 0.95 are shown above the nodes. New isolates are blue, and ex-holotype strains are in bold. The tree is rooted with *Pleurotheciella rivularia* (CBS 125237 and CBS 125238).

To determine the taxonomic placement of the newly collected taxa, phylogenetic analyses were performed using a combined ITS, LSU, SSU and *rpb2* sequence matrix. The dataset included 53 strains within Pleurotheciaceae, with *Pleurotheciella rivularia* (CBS 125237 and CBS 125238) designated as the outgroup (Li et al. 2025). The aligned concatenated matrix comprised 5273 characters, including gaps, with individual gene regions distributed as follows: ITS (1–604 bp), LSU (605–2011 bp), SSU (2012–4092 bp), and *rpb2* (4093–5273 bp). The PHT with heuristic search showed no significant incongruence among individual gene partitions (P = 0.92), supporting the combination of the four loci for phylogenetic reconstruction. Phylogenetic trees generated from ML and BI analyses displayed highly congruent topologies (data not shown), and single-gene analyses also revealed no major conflicts relative to the combined phylogeny (Suppl. material 1: figs S1–S4). The best-scoring ML tree (log-likelihood = -16592.025607) was used to illustrate phylogenetic relationships among taxa, and is presented in Fig. 1. For the BI analysis, the final average standard deviation of split frequencies was 0.009936, indicating sufficient topological convergence.

The phylogenetic analysis (Fig. 1) resolved nine new isolates (GMBCC 2513, GMBCC 2514, GMBCC 2515, GMBCC 2516, GMBCC 2517, GMBCC 2518, GMBCC 2522, GMBCC 2523 and GMBCC 2524) within the *Phaeoisaria* clade. Specifically, *Phaeoisaria bailongtanensis* (GMBCC 2513 and GMBCC 2514) represented an independent monophyletic clade occupying a sister position to *P. loranthacearum* (KUNCC 24-18588) with strong statistical support (100% ML BS/1.0 PP). Strains of *Phaeoisaria magnoliicola* (GMBCC 2523 and GMBCC 2524) formed a distinct lineage as sister to the clade comprising *P. mononematosa* (CGMCC 3.28757) and *P. sparsa* (FMR 11939), with strong statistical support (98% ML BS/1.0 PP). The two strains of *P. qujingensis* (GMBCC 2515 and GMBCC 2516) formed a distinct lineage as sister to *P. obovata* (CGMCC 3.27015 and KUNCC 23-15598) with 56% ML BS and 1.0 PP support. In addition, our three isolates of *P. clematidis* (GMBCC 2517, GMBCC 2518 and GMBCC 2522) clustered with conspecific reference strains (DAOM 226789, MFLUCC 16-1273 and MFLUCC 17-1341) with strong statistical support (100% ML BS/1.0 PP).

### Taxonomy

#### Phaeoisaria bailongtanensis

Taxon classificationFungiXylarialesDiatrypaceae

Q.J. Shang, K.D. Hyde & D.Q. Dai sp. nov.

D2167D42-FCBF-5F2D-B770-A84B2A27055F

Fig. 2

##### Etymology.

The specific epithet “bailongtanensis” refers to Bailongtan Park, where the fungus was collected.

**Figure 2.**
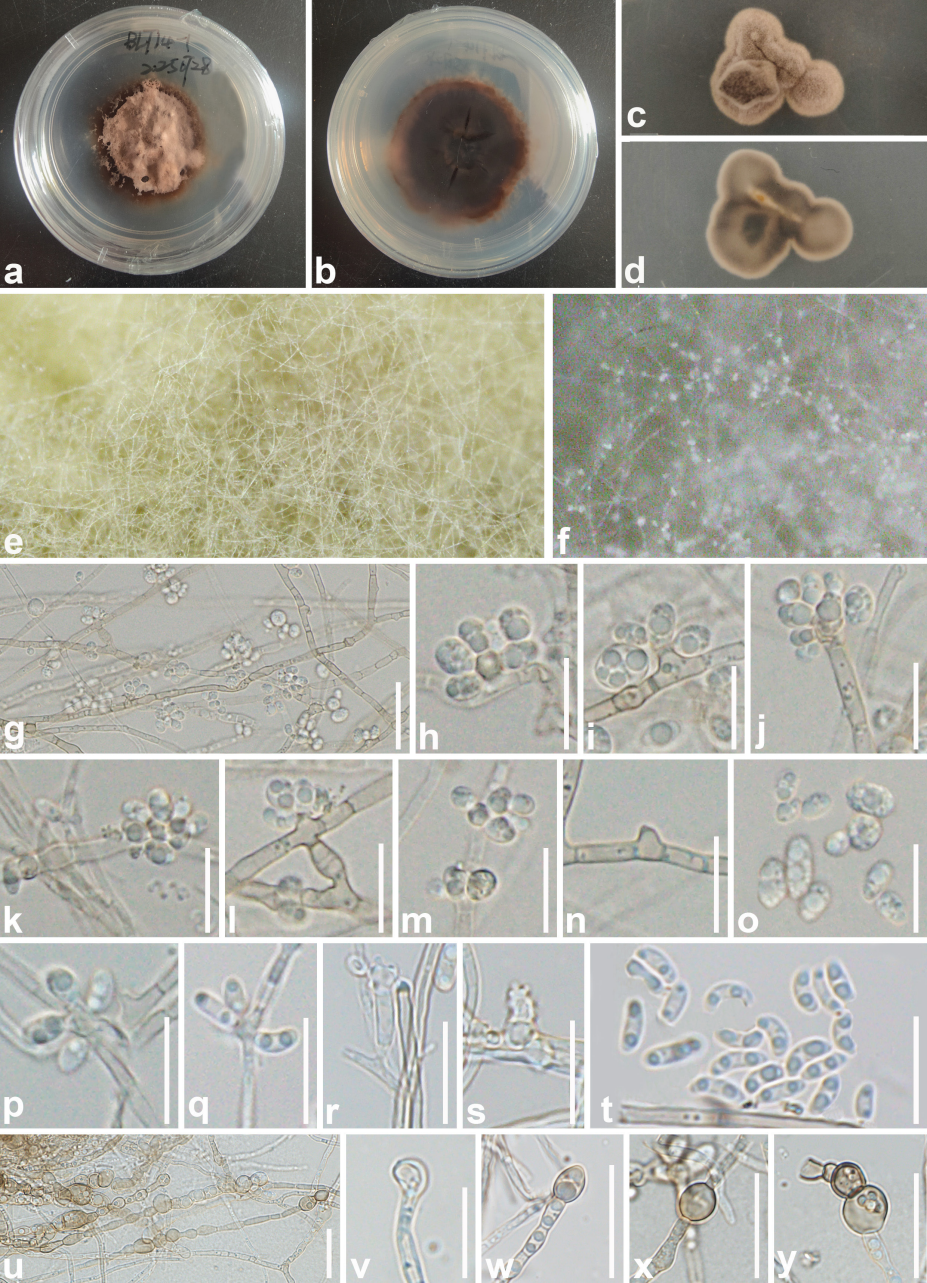
Sporulation observed on PDA of *Phaeoisaria bailongtanensis* (GMBCC 2513, ex-holotype). **a–d**. Colony on PDA from surface and reverse (**a, c**. colony from above; **b, d**. colony from below). **e–g**. Mycelia; **h–m, p–r**. Conidiogenous cells with conidia; **n, s**. Conidiogenous cells; **o, t**. Conidia; **u–y**. Chlamydospores. Scale bars: 20 µm (**g, u–y**); 10 µm (**h–t**).

##### Holotype.

HKAS 154804.

##### Description.

***Saprobic*** on decaying wood of *Magnolia delavayi*. **Sexual morph**: Undetermined. **Asexual morph**: Evanescent on the natural substrate. ***Culture characteristics***: Conidia germinated on PDA within 24 h. Colonies on PDA reaching 30 mm diam. after two weeks at room temperature, raised, umbonate at the centre, with irregular margins; surface velvety, rough to smooth, greyish-brown at the centre, light whitish-grey to brown at the margin; reverse pale brown to dark brown at the centre, whitish-grey to brown at the margin, with distinct concentric pigmentation rings, margin smooth. ***Synnemata*** and ***conidiophores*** are absent. ***Conidiogenous cells*** (2.1–)3.4–5.9(–7.8) × (1–)1.4–2.3(–3) μm (x̄ = 4.7 × 1.9 μm, n = 35), integrated, lateral or intercalary, polyblastic, reduced, doliiform to ampulliform, smooth-walled, hyaline to pale brown, with conidia arising from one to several cylindrical or clavate denticles. ***Conidia*** of two types, type 1: (2.9–)3.6–5.2(–7.2) × (2.3–)2.9–4.4(–5.9) μm (x̄ = 4.4 × 3.6 μm, n = 50), acrogenous or acropleurogenous, globose to subglobose, ellipsoidal, ovoid, aseptate, hyaline, smooth-walled, guttulate; type 2: (3.9–)4.2–5.2(–5.9) × (1.6–)1.7–2.2(–2.7) μm (x̄ = 4.7 × 2 μm, n = 50), solitary, dry, acrogenous or acropleurogenous, allantoid to reniform, obtuse at both ends, aseptate, hyaline, smooth-walled, guttulate. ***Chlamydospores*** (4.9–)5.8–8.2(–9.7) × (3.9–)4.6–6.8(–9.2) μm (x̄ = 7 × 5.7 μm, n = 35), produced from hyphae, intercalary or terminal, solitary or in small clusters, globose to subglobose, thick-walled, smooth, aseptate, pale brown to brown.

##### Material examined.

China • Yunnan Province, Kunming City, Bailongtan Park, on dead wood of *Magnolia delavayi* Franch., 20 June 2025, Q.J. Shang, BLT14A (HKAS 154804, ***holotype***, dried culture; ex-holotype GMBCC 2513) • *Ibid*., BLT14B (GMB-W 1260, ***isotype***, dried culture; ex-isotype GMBCC 2514) • *Ibid*., BLT14A/B (HKAS 154805, ***isotype***, dried culture).

##### Additional GenBank numbers.

*tef1α =*PZ583672 (GMBCC 2513), PZ583673 (GMBCC 2514)

##### Notes.

**Figure 3.**
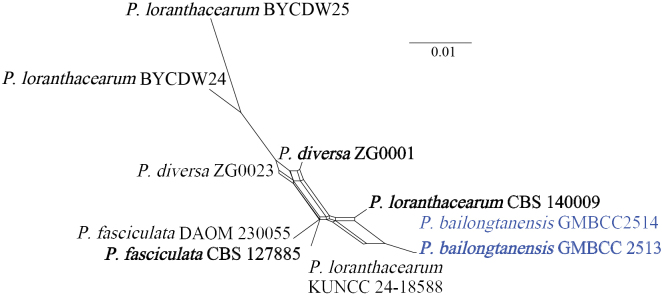
Results of the PHI test for the clade comprising *Phaeoisaria bailongtanensis* and closely related taxa, based on LogDet transformation and split decomposition. PHI test results Φw = 0.3815. The new isolates are blue and ex-holotype strains are in bold.

The phylogenetic analysis (Fig. 1) revealed that our new strains, *Phaeoisaria bailongtanensis* (GMBCC 2513 and GMBCC 2514), form a distinct, well-supported lineage within *Phaeoisaria* s.str., sister to *P. loranthacearum* (KUNCC 24-18588) with strong statistical support (100% ML BS/1.0 PP). They are closely related to *P. diversa* (ZG001 and ZG0023), *P. fasciculata* (CBS 127885 and DAOM 230055) and *P. loranthacearum* (BYCDW24, BYCDW25 and CBS 140009) in a strongly supported clade (100% ML BS/1.0 PP). Sequence divergences of the ITS, LSU and *rpb2* regions between *P. bailongtanensis* and allied taxa are as follows: vs. *P. loranthacearum* CBS 140009 (holotype), 1.14%/1.23% (ITS/LSU); vs. *P. loranthacearum* KUNCC 24-18588, 1.30%/0.12% (ITS/LSU); vs. *P. diversa* ZG001 (holotype), 2.33% (ITS); and vs. *P. fasciculata* CBS 127885 (holotype), 2.20%/1.20%/6.24% (ITS/LSU/*rpb2*) (Crous et al. 2015; Réblová et al. 2016; Luo et al. 2025; Wang et al. 2025). Although sequence comparisons revealed low divergence between *P. bailongtanensis* and *P. loranthacearum*, and the *rpb2* sequence of the latter is unavailable for further comparison, the PHI test (Φw = 0.3815, P > 0.05, Fig. 3) indicated no significant recombination, supporting the reproductive isolation of the new lineage. Morphologically, *P. bailongtanensis* is readily distinguished from its close relatives (*P. diversa*, *P. fasciculata* and *P. loranthacearum*) by its unique dimorphic conidia (Crous et al. 2015; Réblová et al. 2016; Luo et al. 2025; Wang et al. 2025). Despite the ephemeral structures on the natural substrate being unobserved, stable cultural and micromorphological characters, together with phylogenetic divergence, firmly demonstrate that *P. bailongtanensis* represents a distinct new species in *Phaeoisaria*.

#### Phaeoisaria clematidis

Taxon classificationFungiXylarialesDiatrypaceae

(Fuckel) S. Hughes, Canad. J. Bot. 36: 794 (1958)

8B3A96EA-FC07-5C1A-B4FC-105668A32B33

Figs 4, 5

##### Basionym.

*Stysanus clematidis* Fuckel, Jb. nassau. Ver. Naturk. 23–24: 365 (1870) [1869–70].

**Figure 4.**
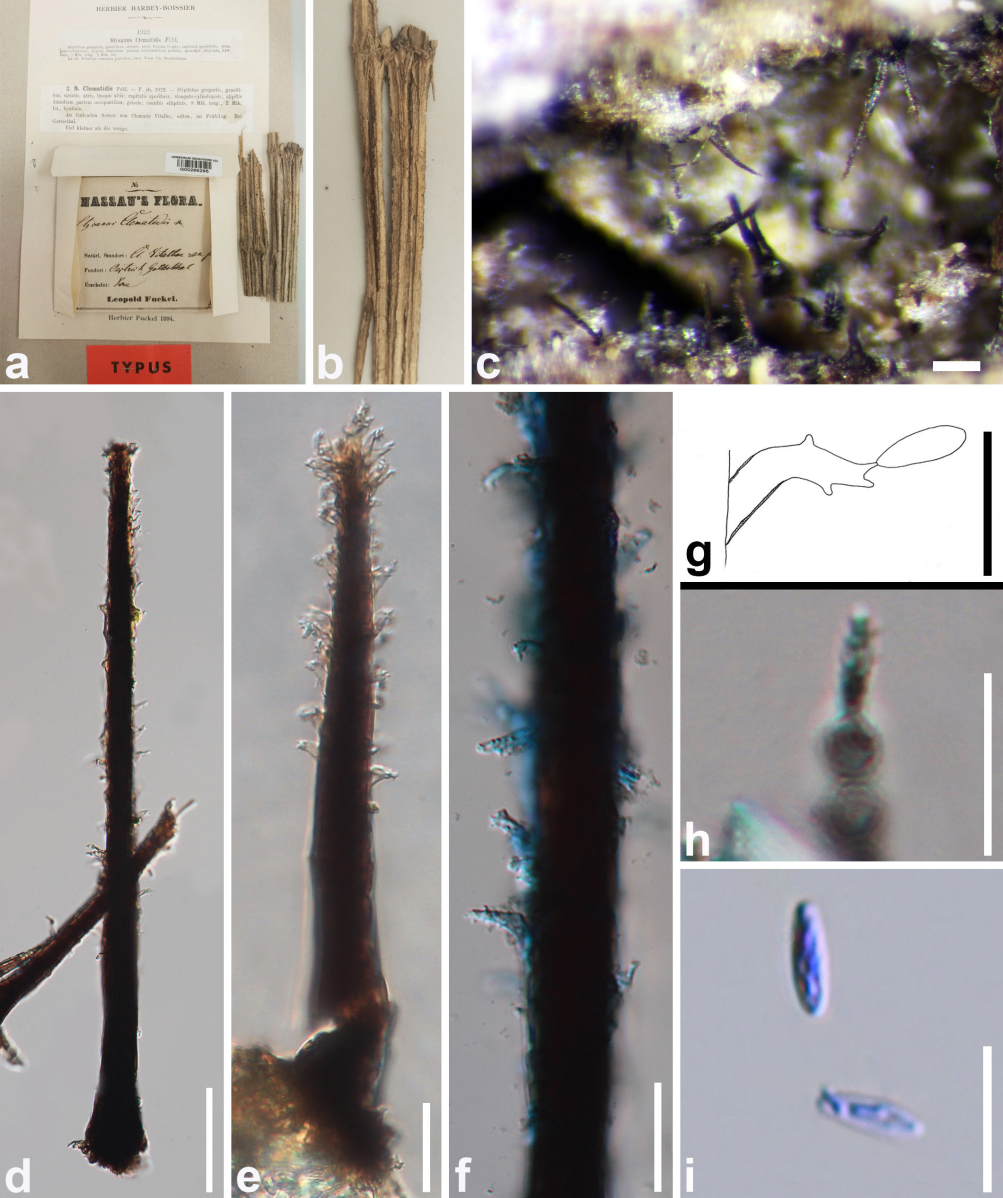
*Phaeoisaria clematidis* (G00266295, Lectotype). **a**. Herbarium material; **b**. Host; **c**. Synnemata on the host; **d, e**. Synnemata; **f**. Conidiophores with conidiogenous cells; **g, h**. Conidiogenous cell with conidia; **i**. Conidia. Scale bars: 100 µm (**c**); 50 µm (**d**); 20 µm (**e**); 10 µm (**f–i**). (Note: **f, h** and **i** were stained with Cotton Blue).

**Figure 5.**
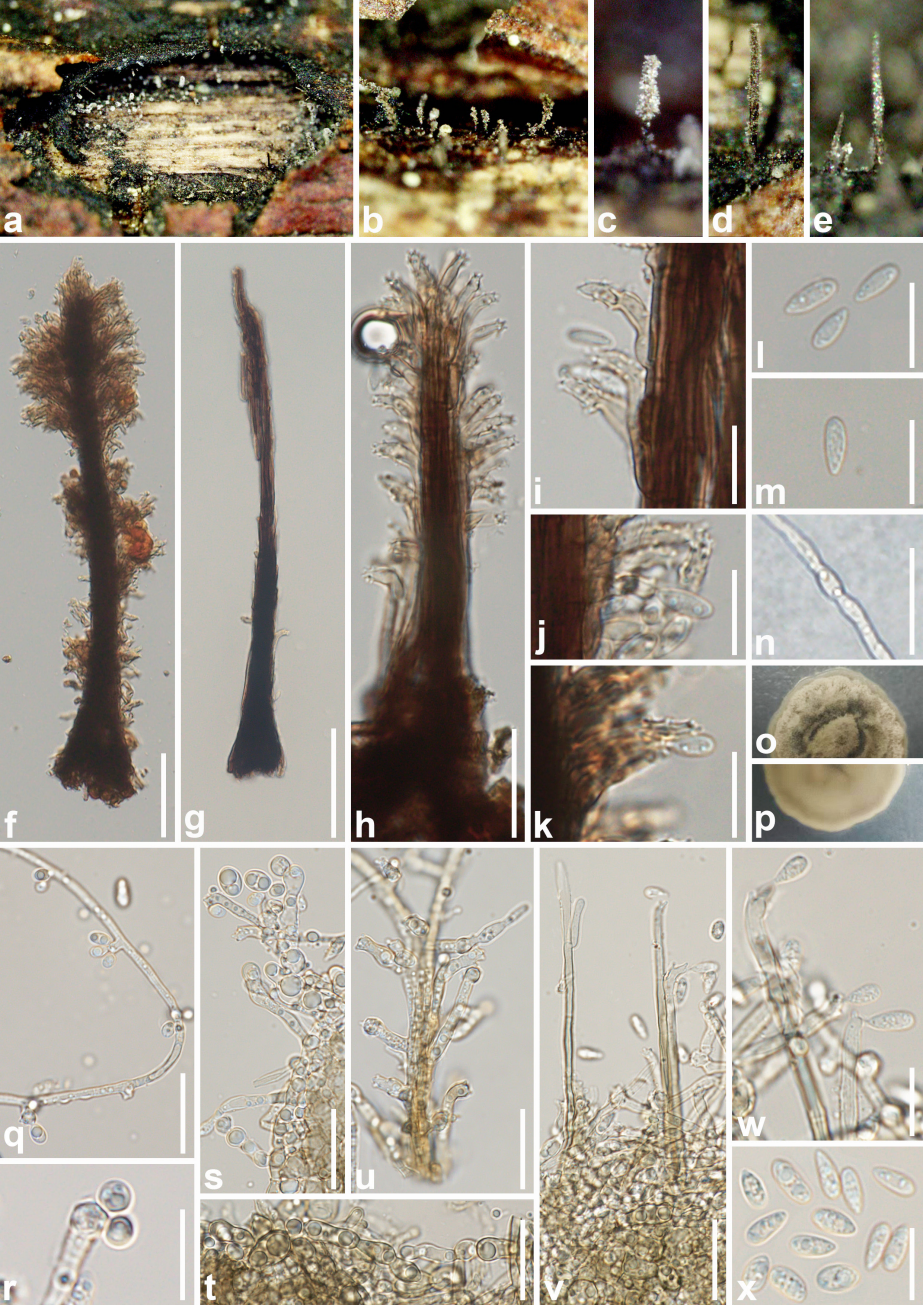
*Phaeoisaria clematidis* (HKAS 154802, GMBCC 2517, reference specimen). **a–m**. Observed from substratum: **a**. Colonies on dead fungal colonies on decaying wood; **b**. Colonies on decaying wood; **c–e**. Synnemata on decaying wood; **f–h**. Synnemata; **i**. Conidiogenous cells; **j, k**. Conidiogenous cells with conidia; **l, m**. Conidia; **n**. Germinating conidium; **o, p**. Colony on PDA from surface and reverse (**o**. colony from above; **p**. colony from below); **q–x**. Sporulation observed on PDA: **q**. Mycelium with conidia; **r–t**. Chlamydospores; **u**. Conidiogenous cells; **v**. Synnemata; **w**. Conidiophores with conidia; **x**. Conidia. Scale bars: 50 µm (**f, g**); 20 µm (**h, n, q, s–v**); 10 µm (**i–m, r, w, x**).

##### Material examined.

***Lectotype designated here***: Germany • Hessen, Oestrich (formerly Nassau), Gottesthal, on rotten branches of *Clematis vitalba*, *Fungi rhenani*, Herbier Fuckel 1894, collection no. 1922, L. Fuckel, (G00266295, syntype of *Stysanus clematidis*) (IF905536) • *Ibid*., ***Isolectotype designated here***: (G00266296, syntype of *Stysanus clematidis*) (IF905537). ***Reference specimen designated here***: China • Yunnan Province, Qujing City, Qujing Normal University, on unidentified dead wood, 6 June 2025, Q.J. Shang, QS69-2 (HKAS 154802, GMB-W 1262), living cultures GMBCC 2517, GMBCC 2518 • *Ibid*., QS69-2B (HKAS 154803, dried culture).

##### Other specimens examined.

China • Yunnan Province, Kunming City, Bailongtan Park, on dead wood of *Magnolia delavayi*, 20 June 2025, Q.J. Shang, BLT14C (HKAS 154806, dried culture), living culture GMBCC 2522.

##### Description.

Based on the lectotype and isolectotype specimens from Germany (G00266295, G00266296), Fig. 4: ***Saprobic*** on rotten branches of *Clematis vitalba*. **Asexual morph**: ***Colonies*** effuse, scattered, dark brown to black, hairy, visible as solitary, dark brown to black conidiophores with white conidia on the upper part. ***Mycelium*** partly immersed, partly superficial, composed of septate, branched, smooth-walled, brown hyphae. ***Synnemata*** 156–355 × 12–21 µm (x̄ = 256 × 16.5 µm, n = 15), erect, rigid, dark brown to black, velvety, smooth, composed of compactly and parallelly appressed conidiophores, with flared conidiogenous cells in the upper half. ***Conidiophores*** macronematous, synnematous, erect, straight, septate, cylindrical, unbranched, smooth-walled, brown to dark brown to black, paler toward the apex. ***Conidiogenous cells*** 7.8–13.5 × 1.7–2.7 µm (x̄ = 10.7 × 2.2 µm, n = 20), integrated, terminal, polyblastic, sympodial, cylindrical, smooth-walled, pale brown to hyaline, straight or slightly flexuous at the base, splaying out with one to several denticulate conidiogenous loci. ***Conidia*** (5.7–)6.7–8.3(–9) × (1–)1.9–2.6(–3.1) µm (x̄ = 7.5 × 2.3 µm, n = 50), solitary, dry, acrogenous, straight, narrowly ellipsoidal or fusiform, obtuse at the ends, aseptate, hyaline, smooth-walled, guttulate.

Based on the fresh specimen from China (HKAS 154802), Fig. 5: ***Saprobic*** on decaying wood; occasionally found on dead fungal colonies associated with woody substrates. **Sexual morph**: Undetermined. **Asexual morph**: ***Colonies*** effuse, aggregated or scattered, dark brown to black, hairy, with brown to dark brown conidiophores bearing white conidia. ***Mycelium*** immersed, composed of septate, branched, brown hyphae. ***Synnemata*** 167–244 × 14–21 µm (x̄ = 206 × 17.3 µm, n = 10), gathered, erect, rigid, cylindrical, brown to dark brown, flared and paler at the apex, composed of compactly and parallelly appressed conidiophores. ***Conidiophores*** macronematous, synnematous, erect, straight or slightly flexuous, septate, cylindrical, unbranched to branched, smooth-walled, brown to dark brown, paler at the apex. ***Conidiogenous cells*** (9.7–)11–16.4(–19.3) × (2–)2.5–3.4(–3.9) μm (x̄ = 13.8 × 2.9 μm, n = 25), integrated, terminal and intercalary, polyblastic, sympodial, cylindrical or tapering towards the tip, smooth-walled, pale brown to hyaline, curved to recurved, longer at the apex of synnema, splaying out with several denticulate conidiogenous loci. ***Conidia*** (5.6–)5.9–6.8(–7.3) × (2.1–)2.3–2.7(–2.9) μm (x̄ = 6.3 × 2.5 μm, n = 30), solitary, dry, acrogenous, straight, fusiform or slightly elongated obovoid, aseptate, hyaline, smooth-walled, guttulate.

##### Culture characteristics.

Conidia germinated on PDA within 24 h, with germ tubes produced from both ends. Colonies on PDA reaching 15 mm diam. after 20 days at room temperature, raised, slightly protruded at the centre, with irregular margins, rough surface, velvety, mycelia dry and dense, pale yellow to dark brown, reverse grey to brownish yellow, smooth from below. ***Synnemata*** 66–97 × 2.4–5.7 µm (x̄ = 81.6 × 4 µm, n = 5), erect, slightly flexuous, hyaline to pale brown, subcylindrical, composed of compactly or loosely appressed conidiophores. ***Conidiophores*** reduced or arising from aerial hyphae, macronematous, mononematous or synnematous, straight or slightly flexuous, cylindrical, branched, smooth-walled, hyaline to brown, paler towards the apex. ***Conidiogenous cells*** 8.8–19 × 2.3–3 µm (x̄ = 14 × 2.6 µm, n = 25), integrated, terminal, polyblastic, sympodial, cylindrical, smooth-walled, hyaline, curved, with several denticulate conidiogenous loci at the expanded apex. ***Conidia*** 6.1–7.5 × 2.5–3.3 µm (x̄ = 6.8 × 2.9 µm, n = 35), solitary, dry, acrogenous, straight, obovoidal, broadly rounded at the apex and minutely truncate at the base, aseptate, hyaline, smooth-walled, guttulate, secession schizolytic. ***Chlamydospores*** 6.9–11 × 4.9–8 µm (x̄ = 9 × 6.4 µm, n = 30), produced from hyphae, catenate, intercalary, lateral to terminal, globose, obovoidal to subglobose, aseptate, hyaline to pale brown, thin, smooth-walled.

##### Additional GenBank numbers.

*tef1α =*PZ583676 (GMBCC 2517), PZ583677 (GMBCC 2518)

##### Notes.

*Phaeoisaria clematidis* (basionym *Stysanus clematidis*) was described by Fuckel (1870) from decaying branches of *Clematis vitalba* L. collected at Gottesthal (Oestrich, Germany), as documented in the exsiccata series Fungi rhenani no. 1922. In the original protologue, Fuckel adopted informal taxonomic terms, referring to synnemata as “stipe” and conidiogenous cells as “spore-bearing caps”, and documented that synnemata were dark and striated, conidiogenous cells were borne on the upper half of synnemata, and conidia were elliptical, hyaline, 8 × 2 μm.

In this study, we re-examined two dried syntype specimens and a fresh collection from terrestrial decaying wood in Yunnan, China. Morphological observations of the syntypes are consistent with the original description: synnemata erect, rigid, dark brown to black, gregarious, slender, composed of compactly parallel hyphae, conidiogenous cells concentrated on the upper half, conidia fusiform, hyaline, (5.7–)6.7–8.3(–9) × (1–)1.9–2.6(–3.1) μm (x̄ = 7.5 × 2.3 μm, n = 50), encompassing the original 8 × 2 μm record. We supplement details not elaborated in the protologue, such as the polyblastic, sympodial conidiogenous cells with denticulate loci and guttulate conidia. The fresh collection is conspecific with the syntypes, exhibiting all essential diagnostic features of *P. clematidis* (e.g., synnematous conidiomata, polyblastic conidiogenous cells, hyaline aseptate conidia), with only slight morphological variations observed. Specifically, the fresh specimen has slightly smaller and paler synnemata (167–244 × 14–21 μm, x̄ = 206 × 17.3 μm, brown to dark brown) compared with the syntypes (156–355 × 15–26 μm, dark brown to black); its conidiogenous cells are larger [(9.7–)11–16.4(–19.3) × (2–)2.5–3.4(–3.9) μm, x̄ = 13.8 × 2.9 μm]; its conidia are shorter and broader [(5.6–)5.9–6.8(–7.3) × (2.1–)2.3–2.7(–2.9) μm, x̄ = 6.3 × 2.5 μm], while the syntypes possess slender conidia.

The morphological characteristics of the re-examined lectotype (G00266295) and isolectotype (G00266296), designated herein, which correspond to the original protologue (Fuckel 1870, Fungi rhenani no. 1922) and two packets cited as “accession no. 2611” in de Hoog and Papendorf (1976), together with those of the fresh reference specimen, are consistent with the original document (Fuckel 1870) and subsequent taxonomic records (Sutton 1973; Deighton 1974; de Hoog and Papendorf 1976; Ellis 1976; Réblová et al. 2016). This confirms the stability of key diagnostic features of *P. clematidis* and provides more accurate quantitative morphological data to complement and refine the gaps in the original and early revised descriptions.

#### Phaeoisaria magnoliicola

Taxon classificationFungiXylarialesDiatrypaceae

Q.J. Shang, J. Yang, K.D. Hyde & D.Q. Dai sp. nov.

6D1E9CD0-1C13-5F4C-8103-148766F8A719

Fig. 6

##### Etymology.

The specific epithet ‘magnoliicola’ refers to the host genus *Magnolia*, from which the holotype was collected.

**Figure 6.**
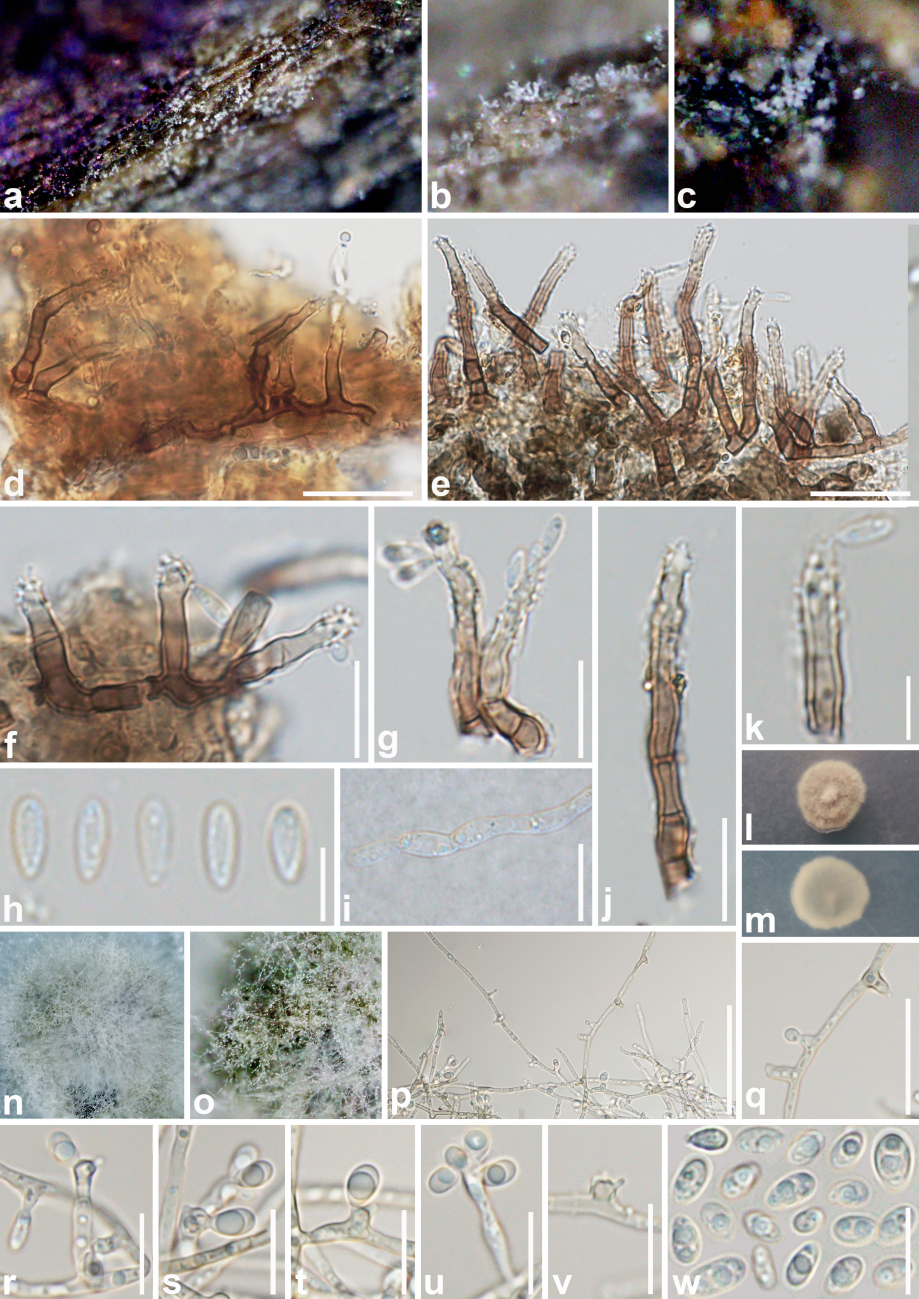
*Phaeoisaria magnoliicola* (HKAS 154807, GMBCC 2523). **a–m**. Observed from substratum: **a–c**. Colonies on the host; **d, e**. Mycelia with conidiophores; **f**. Mycelium with conidiogenous cells; **g, j**. Conidiophores; **k**. Conidiogenous cell with conidium; **h**. Conidia; **i** Germinating conidium; **l, m**. Colony on PDA from surface and reverse (l = colony from above, m = colony from below); **n–w**. Sporulation observed on PDA: **n–p**. Mycelium; **q–u**. Conidiogenous cells with conidia; **v**. Conidiogenous cell; **w**. Conidia. Scale bars: 20 µm (**d, e, q**); 10 µm (**f, g, i, j, r–w**); 5 µm (**h, k**); 50 µm (**p**).

##### Holotype.

HKAS 154807.

##### Description.

***Saprobic*** on decaying wood of *Magnolia delavayi*. **Sexual morph**: Undetermined. **Asexual morph**: ***Colonies*** on natural substrate effuse, scattered, hairy, conidia aggregated at the apex of conidiophores, appearing as white, translucent, tufted or filamentous clusters. ***Mycelium*** mostly immersed, composed of septate, branched, smooth-walled, pale brown to brown hyphae. ***Conidiophores*** 22–33 × 2.5–3.3 µm (x̄ = 27 × 2.9 µm, n = 30), macronematous, mononematous, erect to slightly flexuous, septate, cylindrical, unbranched, smooth-walled, pale brown to brown, hyaline to pale brown toward the apex, thick-walled, solitary or in small groups, arising singly from immersed mycelium, partly reduced to conidiogenous cells. ***Conidiogenous cells*** (6.8–)9.3–19(–26) × (1.9–)2.3–3.1(–3.9) μm (x̄ = 14 × 2.7 μm, n = 30), integrated, terminal, polyblastic, sympodial, cylindrical, smooth-walled, hyaline to pale brown, straight or slightly flexuous, apex slightly swollen, splaying out, with five to multiple small, cylindrical, denticulate conidiogenous loci. ***Conidia*** (3.5–)4.1–5.8(–6.6) × (1.4–)1.6–2(–2.6) μm (x̄ =4.9 × 1.8 μm, n = 30), solitary, dry, acrogenous, straight, ellipsoidal to obovoid, obtuse at apex, truncate at base, aseptate, hyaline, smooth-walled, guttulate, secession schizolytic.

##### Culture characteristics.

Conidia germinating on PDA within 24 h. Colonies on PDA reaching 4 mm diam. after one week at room temperature, raised, umbonate at the centre, with irregular margins; surface velvety to floccose, rough, brittle, greyish-brown at the centre, light whitish-grey at the margin. Reverse pale grey to dark greyish at the centre, whitish-grey at the margin, smooth. ***Conidiogenous cells*** integrated, terminal, polyblastic, sympodial, cylindrical, smooth-walled, hyaline, with one to five denticulate conidiogenous loci at the apex. ***Conidia*** (3.4–)4.7–6.1(–6.7) × (2.4–)3–4.1(–4.7) μm (x̄ =5.4 × 3.6 μm, n = 55), acrogenous, straight, ellipsoidal to obovoidal, broadly rounded at the apex, truncate at base, aseptate, hyaline, smooth-walled, guttulate.

##### Material examined.

China • Yunnan Province, Kunming City, Bailongtan Park, on dead wood of *Magnolia delavayi* Franch., 20 June 2025, Q.J. Shang, BLT14D1 (HKAS 154807, ***holotype***; ex-holotype GMBCC 2523) • *Ibid*., BLT14D2 (GMB-W 1271, ***isotype***; ex-isotype GMBCC 2524) • *Ibid*., BLT14D1A (HKAS 154808, ***isotype***, dried culture).

##### Notes.

**Figure 7.**
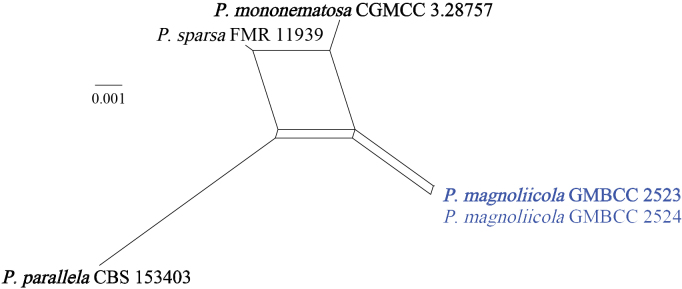
Results of the PHI test for the clade comprising *Phaeoisaria magnoliicola* and closely related taxa, based on LogDet transformation and split decomposition. PHI test results Φw = 0.7142. The new isolates are blue and ex-holotype strains are in bold.

The multi-gene phylogenetic analysis (Fig. 1) revealed that our strains of *Phaeoisaria magnoliicola* (GMBCC 2523, GMBCC 2524) formed a distinct monophyletic clade, as sister to *P. mononematosa* (CGMCC 3.28757) and *P. sparsa* (FMR 11939) with strong statistical support (98% ML BS/1.0 PP). Sequence divergences in ITS, LSU, SSU and *rpb2* between *P. magnoliicola* (HKAS 154807, holotype) and related taxa are as follows: 0%/0.2%/1.07%/0.97% vs. *P. sparsa* (FMR 11939); 0.45%/0%/1.01%/1.26% vs. *P. mononematosa* (CGMCC 3.28757, holotype); and 1.03%/0.24%/0.86%/1.43% vs. *P. parallela* (CBS 153403, holotype). Although the ITS and LSU sequences showed low divergence from closely related species, *P. magnoliicola* is morphologically distinct in being non-synnematous and producing simple, short, and thick-walled conidiophores and small, aseptate, obovoid conidia with a truncate base. In contrast, *P. sparsa* possesses well-developed (typical) synnemata and large, fusiform conidia that are often 0–3-septate (Sutton 1973; Réblová et al. 2025); *P. mononematosa* is characterised by longer conidiophores, fusiform-ellipsoidal conidia with obtuse ends, and has so far been reported exclusively from submerged wood, whereas *P. magnoliicola* was isolated as a saprobe on terrestrial decaying wood of *Magnolia* sp. (Wang et al. 2025). *Phaeoisaria parallela*, meanwhile, forms atypical synnemata (loosely organized, parallelly arranged conidiophores that are not tightly appressed, Réblová et al. 2025). Combined with the phylogenetic distinctiveness and absence of significant recombination (PHI test, P = 0.7142 > 0.05, Fig. 7), these morphological and ecological discontinuities confirm that our isolates represent a new species.

#### Phaeoisaria qujingensis

Taxon classificationFungiXylarialesDiatrypaceae

Q.J. Shang, K.D. Hyde, Wijayaw. & D.Q. Dai sp. nov.

FB1AF51B-B482-5C3F-90B3-A00E5B2D4F24

Figs 8, 9

##### Etymology.

The specific epithet refers to Qujing City, where the fungus was collected.

**Figure 8.**
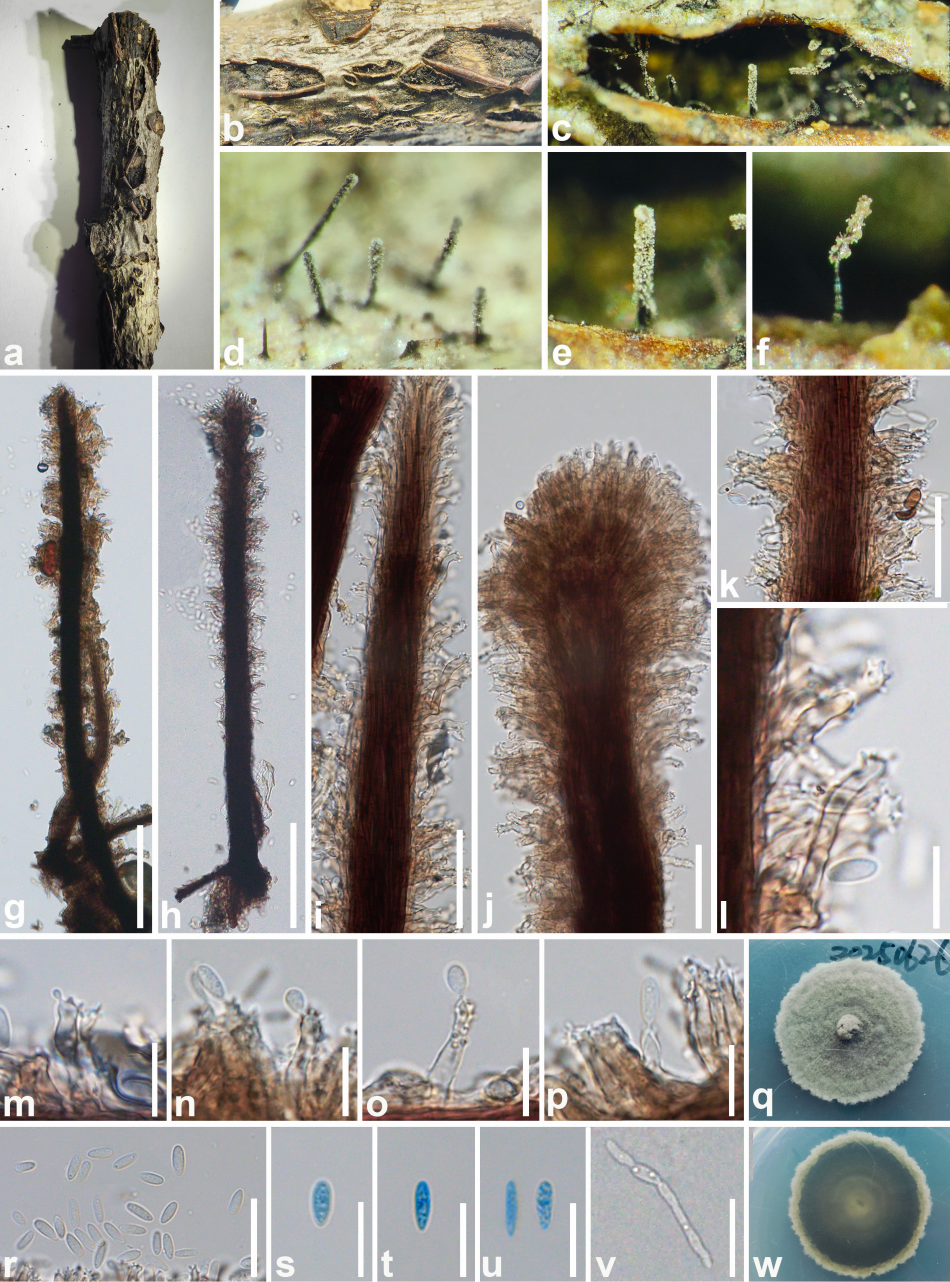
Asexual morph of *Phaeoisaria qujingensis* (HKAS 154800, holotype). **a, b**. Host; **c, e**. Synnemata on dead fungal colonies on decaying wood; **d, f**. Synnemata on decaying wood; **g–j**. Synnemata; **k, l**. Conidiogenous cells; **m–p**. Conidiogenous cells with conidia; **q, w**. Culture characteristic on PDA (**q** colony from above; **w** colony from below); **r–u**. Conidia; **v**. Germinating conidium. Scale bars: 100 µm (**g, h**); 30 µm (**j–k**); 20 µm (**r, v**); 10 µm (**l–p, s–u**). (note: **s–u** were stained with Cotton Blue).

**Figure 9.**
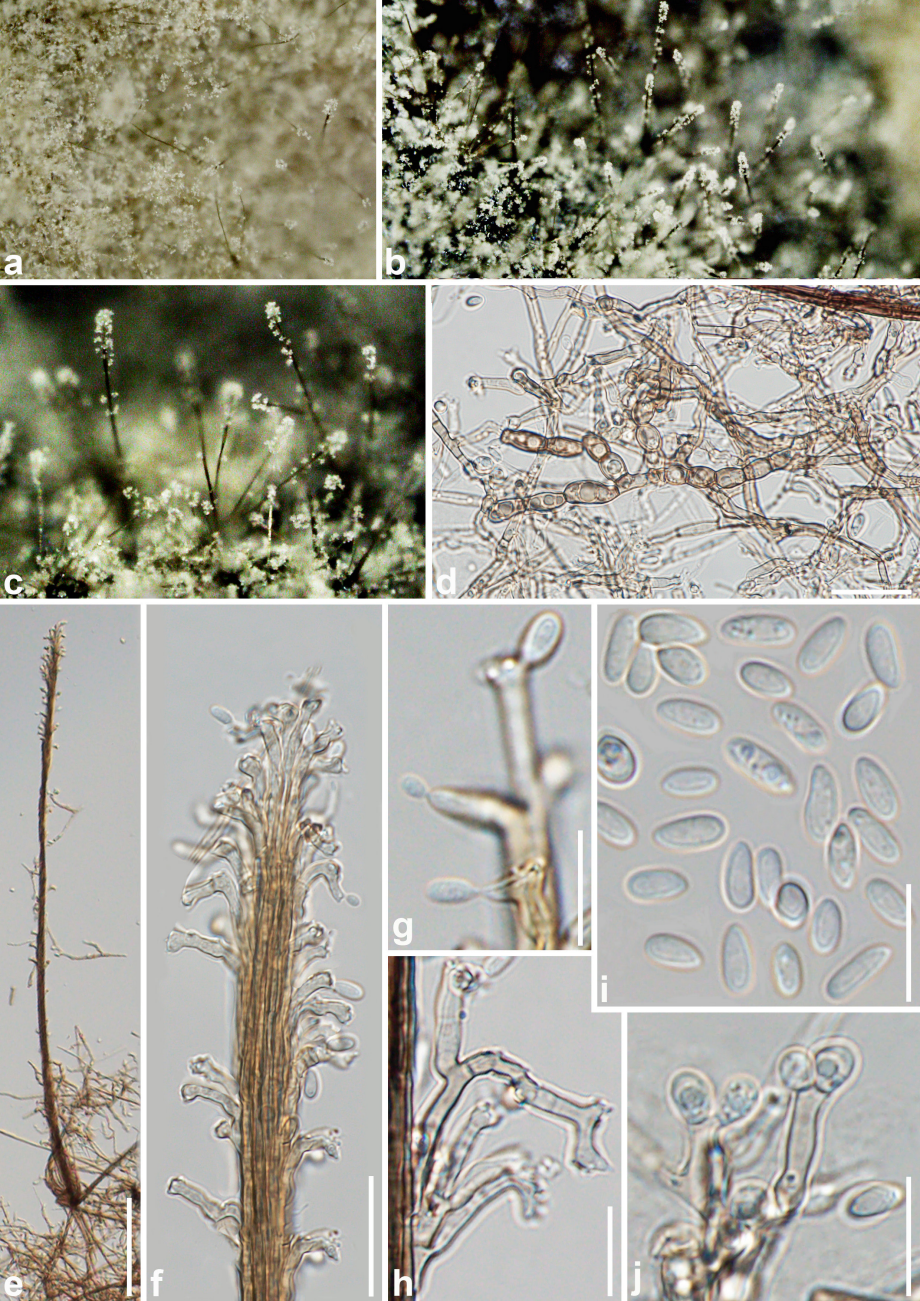
Sporulation observed of *Phaeoisaria qujingensis* on PDA (GMBCC 2515). **a**. Colonies; **b, c, e** Synnemata; **d**. Chlamydospores; **f**. Apex of synnema; **g**. Conidiogenous cells with conidia; **h**. Conidiogenous cells; **i**. Conidia; **j**. Conidiogenous cells with chlamydospores. Scale bars: 20 µm (**d, f, h**); 100 µm (**e**); 10 µm (**g, i, j**).

##### Holotype.

HKAS 154800.

##### Description.

***Saprobic*** on decaying wood, occasionally found on dead fungal colonies associated with woody substrates. **Sexual morph**: Undetermined. **Asexual morph**: ***Colonies*** effuse, scattered, dark brown to black, hairy, with solitary, brown to dark brown conidiophores bearing white conidial masses. ***Mycelium*** partly immersed, partly superficial, composed of septate, branched, brown hyphae. ***Synnemata*** 339–736 μm high, 21.5–43.5 μm diam. (x̄ = 536 × 32.5 μm, n = 10), aggregated, erect, rigid, cylindrical to clavate, brown to dark brown, flared and paler at the apex, composed of compactly and parallelly appressed conidiophores. ***Conidiophores*** macronematous, synnematous, erect, straight or slightly flexuous, septate, cylindrical, unbranched to branched, smooth-walled, brown to dark brown, paler at the apex. ***Conidiogenous cells*** (11–)14–21(–24) × (1.8–)2.2–3.2(–3.9) μm (x̄ = 17.7 × 2.7 μm, n = 25), integrated, terminal and intercalary, polyblastic, sympodial, cylindrical or tapering toward the tip, smooth-walled, pale brown to hyaline, curved to recurved, longer at the apex of synnema, splaying out with several denticulate conidiogenous loci. ***Conidia*** (5–)6.3–7.7(–8.3) × (1.6–)2.3–3(–3.5) μm (x̄ = 7 × 2.6 μm, n = 55), solitary, dry, acrogenous, fusiform or elongated obovoid, rounded at the apex and obtuse at the base, aseptate, hyaline, smooth-walled, guttulate.

##### Culture characteristics.

Conidia germinating on PDA within 12 h, with germ tubes produced from both ends. Colonies on PDA reaching 20 mm in diameter after 4 weeks at room temperature, with slightly raised centre, irregular margins, rough surface, mycelia dry and dense; yellowish to grey, dark brown to black at centre, reverse white to yellowish at margin, smooth. ***Synnemata*** 356–525 × 6.8–12.5 µm (x̄ = 440 × 9.6 µm, n = 30), erect, slightly flexuous, dark brown to black, subcylindrical, composed of compactly and parallelly appressed conidiophores. ***Conidiophores*** reduced or arising from aerial hyphae, macronematous, mononematous or synnematous, straight or slightly flexuous, cylindrical, branched, smooth-walled, dark brown to black, paler towards the apex. ***Conidiogenous cells*** (7.8–)9.1–15.5(–18.8) × (1.7–)2.1–2.7(–3.1) μm (x̄ = 12.3 × 2.4 μm, n = 25), integrated or discrete, terminal, polyblastic, sympodial, cylindrical, smooth-walled, hyaline, curved, branched, with several denticulate conidiogenous loci at the expanded apex. ***Conidia*** (4.8–)5.5–7(–7.6) × (2.1–)2.3–2.9 (–3.3) μm (x̄ = 6.2× 2.6 μm, n = 50), solitary, dry, acrogenous, straight, obovoidal, broadly rounded at the apex, aseptate, hyaline, smooth-walled, guttulate, secession schizolytic. Chlamydospores (4.8–) 7.1–10.5 (–12.3) × (4.5–) 5.6–8.7 (–10.6) μm (x̄ = 8.8× 7.2 μm, n = 40), produced from hyphae, catenate, intercalary, lateral to terminal, globose, obovoidal to subglobose, aseptate, hyaline to pale brown, thin-walled, smooth.

##### Material examined.

China • Yunnan Province, Qujing, Qujing Normal University, on unidentified dead wood, 6 June 2025, Q.J. Shang, QS44-5 (HKAS 154800, ***holotype***; ex-holotype GMBCC 2515) • *Ibid*., (GMB-W 1261, ***isotype***; ex-isotype GMBCC 2516) • *Ibid*., QS44-5A (HKAS 154801, ***isotype***, dried culture).

##### Additional GenBank numbers.

*tef1α =*PZ583674 (GMBCC 2515), PZ583675 (GMBCC 2516).

##### Notes.

**Figure 10.**
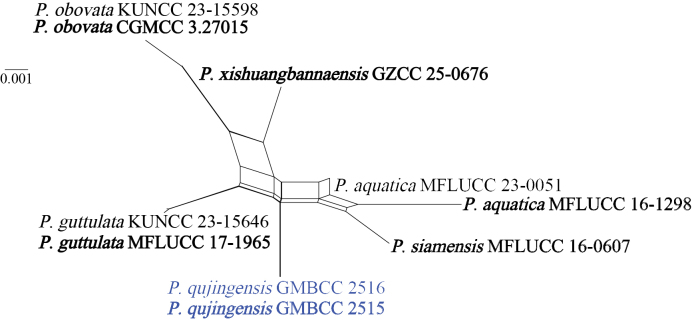
Results of the PHI test for the clade comprising *Phaeoisaria qujingensis* and closely related taxa, based on LogDet transformation and split decomposition. PHI test results Φw = 0.9934. The new isolates are blue and ex-holotype strains are in bold.

The phylogenetic inference obtained in this study (Fig. 1) showed that the new species *Phaeoisaria qujingensis* (GMBCC 2515 and GMBCC 2516) formed a distinct lineage, sister to *P. obovata* (CGMCC 3.27015 and KUNCC 23-15598) and was closely related to *P. aquatica* (MFLUCC 16-1298 and MFLUCC 23-0051), and *P. siamensis* (MFLUCC 16-0607). Sequence divergence analyses confirm its interspecific distinctness from allied species, with genetic distances exceeding intraspecific variation thresholds: 2.11% (ITS), 1.51% (*rpb2*) vs. *P. aquatica* (MFLUCC 16-1298, holotype), 6.43% (ITS), 2.71% (*rpb2*) vs. *P. obovata* (HKAS 131983, holotype), 1.32% (ITS), 1.07% (LSU), 2.91% (*rpb2*) vs. *P. siamensis* (MFLU 16-0953, holotype). A PHI test detected no significant recombination (Φw = 0.9934, P > 0.05, Fig. 10) within the lineage, providing additional support for recognizing *P. qujingensis* as a distinct species. Morphologically, *P. qujingensis* is consistent with the generic concept of *Phaeoisaria* (de Hoog and Papendorf 1976; Réblová et al. 2016). It can be distinguished from the three related species (*P. aquatica*, *P. obovata*, and *P. siamensis*) by several key morphological characters (Luo et al. 2018; Hyde et al. 2019; Wang et al. 2024; Table 2): Notably, it possesses robust synnemata (339–736 × 21.5–43.5 μm), which are clearly broader than those of *P. aquatica*, *P. obovata*, and *P. siamensis*; conidia of *P. qujingensis* are fusiform to elongated obovoid, differing from more globose conidia of the other three species (Luo et al. 2018; Hyde et al. 2019; Wang et al. 2024). Therefore, the phylogenetic and morphological evidence strongly supports the recognition of *P. qujingensis* as a new species.

**Table 2.** Standardized comparison of morphological characteristics of *Phaeoisaria* species. Dimensions are given as length × width (µm).

**Taxa**	**Synnemata/Conidiophores**	**Conidiogenous cells**	**Conidia**	**References**
*Phaeoisaria aquatica* (MFLU 17–0918, holotype)	Synnematous, Synnemata: 1028–1262 × 3.5–4.5 ( x̄= 1145 × 4, SD = 117/0.5, n = 10)	–	Ellipsoidal to obovoid, 6.5–7.5 × 2.5–3.5 (x̄ = 7 × 3, SD = 0.5/0.5, n = 45)	Luo et al. (2018)
*P. bailongtanensis* (HKAS 154804, holotype)	Non-synnematous, Conidiophores absent.	(2.1–)3.4–5.9(–7.8) × (1–)1.4–2.3(–3) (x̄ = 4.7 × 1.9, n = 35)	Globose to subglobose, ellipsoidal, ovoid, (2.9–) 3.6–5.2 (–7.2) × (2.3–)2.9–4.4(–5.9) (x̄ = 4.4 × 3.6, n = 50); allantoid to reniform, (3.9–) 4.2–5.2 (–5.9) × (1.6–)1.7–2.2(–2.7) (x̄ = 4.7 × 2, n = 50)	This study
*P. clematidis* (G00266295, Lectotype)	Synnematous, Synnemata: 156–355 × 12–21 (x̄ = 256 × 16.5, n = 15)	7.8–13.5 × 1.7–2.7 (x̄ = 10.7 × 2.2, n = 20)	Fusiform, (5.7–)6.7–8.3(–9) × (1–)1.9–2.6(–3.1) (x̄ = 7.5 × 2.3, n = 50)	This study
*P. diversa* (HHAUF 170532, holotype)	Non-synnematous, Conidiophores: 5.0–14.5 × 2.0–4.0	4.5–9.0 × 4.0–13.0	Ellipsoidal or long oval, 3.5–6.8 × 1.5–2.8	Luo et al. (2025)
*P. fasciculata* (PRM 933855, holotype)	Non-synnematous, Conidiophores: fasciculate, 25–65 × 3.0–3.5	10–29(–36) × 2.5–3.5(–4.0) (x̄ = 20.2 × 3.1, n = 30)	Ellipsoidal to obovoid, 6.0–8.0 × 2.0	Réblová et al. (2016)
*P. guttulata* (MFLU 18-0139, holotype)	Synnematous, Conidiophores: 480–700 × 2–5	–	Globose to obovoid, 3.5–5.5 × 2.5–4.8 (x̄ = 4.5 × 3.5, n = 20)	Hyde et al. (2018)
*P. loranthacearum* (CBS H-22261, holotype)	Non-synnematous, Conidiophores: 10–30 × 2–3	7–16 × 2–3.5	Fusoidal-ellipsoidal, (5)7–8(9) × (1.5)2(3)	Crous et al. (2015)
*P. loranthacearum* (HKAS 146439)	Non-synnematous, Conidiophores: 25–69 × 2–3.2 (x̄ = 51.2 × 2.6, n = 30)	–	Ellipsoidal to fusiform 4.9–7.1 × 1.6–2.7 (x̄ = 6.1 × 2, n = 30)	Wang et al. (2025)
*P. mononematosa* (HKAS 146443, holotype)	Non-synnematous, Conidiophores: 29–81 × 1.4–2.7 (x̄ = 48.1 × 2, n = 20),	–	Fusiform to ellipsoidal, 4.4–7.1 × 1.6–2.2 (x̄ = 5.7 × 1.9, n = 20)	Wang et al. (2025)
*P. magnoliicola* (HKAS 154807, holotype)	Non-synnematous, Conidiophores: 22–33 × 2.5–3.3 (x̄ = 27 × 2.9, n = 30)	(6.8–)9.3–19(–26) × (1.9–)2.3–3.1(–3.9) (x̄ = 14 × 2.7, n = 30)	Ellipsoidal to obovoid, (3.5–)4.1–5.8(–6.6) × (1.4–)1.6–2(–2.6) (x̄ = 4.9 × 1.8, n = 30)	This study
*P. obovata* (HKAS 131983, holotype)	Synnematous, Synnemata: 150–1370 × 7.1–21 ( x̄ = 727.5 × 14.6, n = 10)	7.6–22 × (1.2–)1.5–2.3(–2.8) (x̄ = 14.3 × 2, n = 20)	Subglobose to obovoid to elongated obovoid, 3.7–7.9 × 2–3.2 (x̄ = 5.6 × 2.5, n = 40)	Wang et al. (2024)
*P. parallela* (PRA-22501, holotype)	Synnematous, Synnemata: 70 × 10–14	(9.5–)11–15.5(–19) × 2.5–3.5	Fusiform to oblong, (6.5–)7–9(–10) × 1.5–2 (mean ± SD = 7.9 ± 0.7 × 1.8 ± 0.2)	Réblová et al. (2025)
*P. qujingensis* (HKAS 154800, holotype)	Synnematous, Synnemata: 339–736 × 21.5–43.5 (x̄ = 536 × 32.5)	(11–)14–21(–24) × (1.8–)2.2–3.2(–3.9) (x̄ = 17.7 × 2.7)	Fusiform or elongated obovoid, (5–)6.3–7.7(–8.3) × (1.6–)2.3–3(–3.5) (x̄ = 7 × 2.6)	This study
*P. siamensis* (MFLU 16-0953, holotype)	Synnematous, Synnemata: 330–380 × 20–25(–30) (x̄ = 360 × 24.5, n = 20)	8–12 × 2–2.5 (x̄ = 10.5 × 2.3, n = 20)	Globose to subglobose, 5–8 × 3–4 (x̄ = 6.2 × 3.5, n = 30)	Hyde et al. (2019)
*P. sparsa* (FMR 11939)	Synnematous, Synnemata: 220–250 × 17–28	15–20.5 × 3–3.5	Fusiform to oblong-clavate, 0–3-septate, 12–17 × 2.5–3.5	Réblová et al. (2025)
*P. xishuangbannaensis* (GZAAS25-0706, holotype)	Synnematous, Synnemata: 474–521 × 19–32 µm (x̄ = 505 × 24, n = 15)	5.5–14.5 × 1.5–3 (x̄ = 9 × 2.5, n = 25)	Ellipsoidal to oval, 4.5–7 × 2–3 (x = 6 × 2.5, n = 20)	Tian et al. (2026)

Isolated en-dash (–) = missing data; en-dashes between numbers represent measurement ranges.

## Discussion

*Phaeoisaria* comprises saprobic hyphomycetes characterized by macronematous to semi-macronematous conidiophores, polyblastic conidiogenous cells, hyaline aseptate conidia, and synnemata in most species (Réblová et al. 2016; Lin et al. 2025). Based on morphological characteristics and multi-gene (ITS, LSU, SSU, *rpb2*) phylogenetic analyses, three new species, *Phaeoisaria bailongtanensis*, *P. magnoliicola* and *P. qujingensis*, are introduced from decaying wood in Yunnan, China. Herein, we comprehensively delimit these three new species by integrating their phylogenetic positions and morphological characteristics, and discuss the taxonomic issues, ecological implications and future research directions of the genus.

### Phylogenetic position and morphological delimitation of the new species

The concatenated ML/BI multi-gene phylogenetic tree (Fig. 1) supports the placement of *Phaeoisaria bailongtanensis*, *P. magnoliicola* and *P. qujingensis* within *Phaeoisaria* s.str., with all forming independent clades distinct from all known congeners.

*Phaeoisaria bailongtanensis* nested within a subclade containing *P. diversa*, *P. fasciculata* and *P. loranthacearum*, *P. magnoliicola* within the clade closely related to the *P. mononematosa*, *P. parallela* and *P. sparsa* lineage. These together constitute a morphologically divergent, predominantly non-synnematous group separated from the genus’s typical synnematous lineages. Morphologically, *P. bailongtanensis* produces dimorphic conidia, which distinguishes it from species (*P. diversa*, *P. fasciculata* and *P. loranthacearum*) in the sister clade (Crous et al. 2015; Réblová et al. 2016; Xu et al. 2020; Luo et al. 2025; Wang et al. 2025). *Phaeoisaria magnoliicola* can be distinguished from closely related clades (*P. mononematosa*, *P. parallela*, *P. sparsa*) by its non-synnematous, short conidiophores, and small obovoid conidia (see notes and Table 2 for detailed data; Sutton 1973; Réblová et al. 2025; Wang et al. 2025).

*Phaeoisaria qujingensis* clusters with the typical synnematous species *P. aquatica*, *P. obovata* and *P. siamensis*, with *P. guttulata* and *P. xishuangbannaensis* as their closest relatives, and this clade is phylogenetically distant from the non-synnematous clade containing *P. magnoliicola*. *Phaeoisaria qujingensis* can be distinguished from *P. aquatica*, *P. guttulata*, *P. obovata*, *P. siamensis*, and *P. xishuangbannaensis* by a combination of core morphological traits (see notes and Table 2 for detailed data; Luo et al. 2018; Hyde et al. 2018, 2019; Wang et al. 2024; Tian et al. 2026): it produces short and broad typical synnemata (vs. slender, variable or longer synnemata in the relatives, including synnemata in *P. xishuangbannaensis*), fusiform to elongated obovoid conidia (vs. smaller, globose or elliptical conidia in the relatives, including globose to obovoid conidia in *P. guttulata*).

Although *P. bambusae* is the nomenclatural type of *Phaeoisaria*, *P. clematidis* has long served as a well-documented reference species for circumscribing the genus, owing to its detailed morphology, accessible type material and clear phylogenetic representativeness (Seifert et al. 2011; Réblová et al. 2016, 2025). *Phaeoisaria* was established by von Höhnel (1909) with the sole species *P. bambusae*, originally regarded as the asexual morph of *Neopeckia bambusae* based on the close association of synnemata and ascomata (Réblová et al. 2016). *Stysanus clematidis* was described by Fuckel (1870) and later transferred to *Phaeoisaria* by Hughes (1958), yielding the currently accepted name *P. clematidis* (Fuckel) S. Hughes. Ellis (1971) treated *P. bambusae* as a synonym of *P. clematidis*, a view supported by Deighton (1974) and de Hoog and Papendorf (1976) based on examinations of type and herbarium specimens. Furthermore, de Hoog and Papendorf (1976) treated *P. clematidis* as the type species of *Phaeoisaria*, criticizing the poor quality of the *P. bambusae* type material and using *P. clematidis* to circumscribe the generic characteristics. Nevertheless, the traditional species concept of *P. clematidis* likely represents a complex of several phylogenetic species (Réblová et al. 2016).

Re-examination of *P. clematidis* type specimens (lectotype and isolectotype designated herein) confirmed its diagnostic traits (fusiform or slightly elongated obovoid conidia with obtuse apex, denticulate conidiogenous cells), establishing a modern morphological benchmark for the genus. All new species are clearly distinct from *P. clematidis* (see Table 2 for detailed data): *Phaeoisaria bailongtanensis* entirely lacks synnemata and conidiophores and produces globose to subglobose, allantoid to reniform conidia, whereas *P. clematidis* forms synnemata and conidiophores in culture, and produces obovoidal conidia that are broadly rounded at the apex and truncate at the base (Figs 2, 5). *Phaeoisaria magnoliicola* has a non-synnematous growth and smaller elliptical-obovoid conidia (vs. typical synnemata and fusiform conidia in *P. clematidis*); *P. qujingensis* is characterized by larger synnemata and conidiogenous cells than those of *P. clematidis*. The morphological divergence thus confirms these species are not conspecific with *P. clematidis*.

### Taxonomic Issues of *Phaeoisaria*

Phylogenetic analysis revealed significant morphological differentiation and taxonomic limitations in *Phaeoisaria*. The *P. bailongtanensis*- and *P. magnoliicola*-affiliated clade in Fig. 1 is characterized by predominantly non-synnematous species with a few atypical synnematous taxa (*P. fasciculata*, *P. parallela*), challenging the traditional view of *Phaeoisaria* as primarily synnematous (Réblová et al. 2025). This synnematal variation likely reflects ecological adaptation and provides a new perspective for the classification. Additionally, *P. fasciculata* and *P. loranthacearum* (recently synonymized by Wang et al. 2025) are retained as separate names in our tree, reflecting long-standing taxonomic uncertainty in this group. The *P. loranthacearum* and *P. fasciculata* clade (including *P. bailongtanensis*) also has low phylogenetic resolution, due to the lack of protein-coding genes (*rpb2*, *tef1α*) in most strains (only ITS/LSU sequences available). Protein-coding genes such as *rpb2* are critical for resolving closely related *Phaeoisaria* species (Réblová et al. 2016), and the slow evolutionary rate of ribosomal genes fails to distinguish morphologically conserved congeners, highlighting a major limitation of current *Phaeoisaria* (at least in the *P. bailongtanensis*- and *P. magnoliicola*-affiliated clade) taxonomy: most described species are based solely on morphology or partial ribosomal data, lacking robust multi-gene phylogenetic support (Réblová et al. 2016; Wang et al. 2025).

Although stable dimorphic conidia in culture were previously considered rare in *Phaeoisaria*, with only *P. sedimenticola* reliably documented (Cheng et al. 2014), the discovery of *P. bailongtanensis* in this study represents the second confirmed species with this trait. This indicates that dimorphic conidia are not an isolated exception within the genus but may represent an important taxonomic character with diagnostic value, potentially reflecting more complex morphological diversification and evolutionary differentiation within *Phaeoisaria*.

### Ecological implications and future research prospects

The three new species expand the known diversity of *Phaeoisaria* in subtropical forest ecosystems of Yunnan, providing fundamental data for the genus’s ecological distribution research. Globally, *Phaeoisaria* species are diverse in freshwater and karst habitats (particularly submerged wood in streams, lakes and karst areas) and terrestrial species are common in temperate and tropical forests, with recent discoveries extending their distribution to high-altitude environments (e.g., *P. motuoensis* and *P. yadongensis*) (Luo et al. 2018; Boonmee et al. 2021; Liu et al. 2022; Yang et al. 2023; Xu et al. 2024; Li et al. 2025; Wang et al. 2025). China has emerged as a key diversity centre for the genus, with 18 documented species predominantly associated with decaying wood in freshwater and terrestrial habitats (Luo et al. 2018, 2019; Liu et al. 2022; Li et al. 2025; Luo et al. 2025; Wang et al. 2025; Zhang et al. 2025; Tian et al. 2026), and our new species further enriches the overall diversity of this genus.

Specimens of *Phaeoisaria clematidis* and *P. qujingensis* obtained in this study were occasionally found colonising dead fungal colonies on decaying wood (Figs 5a, 8c, e). This specialised ecological niche has never previously been documented in *Phaeoisaria* or its affiliated family. It suggests potential mycoparasitic or symbiotic life strategies within the genus, and reflects complex nutritional interactions among wood-inhabiting fungal communities that warrant further investigation. Furthermore, three or more *Phaeoisaria* species, represented by five isolates (*P. bailongtanensis*GMBCC 2513, GMBCC 2514; *P. clematidis*GMBCC 2522; *P. magnoliicola*GMBCC 2523, GMBCC 2524; Fig. 1) were recovered from a single sample (BLT14), where the sub-serial numbers (BLT14A/B, BLT14C, and BLT14D1/D2) correspond exactly to the three aforementioned species, respectively. Additional colonies were also observed but not isolated or identified, leaving open the possibility that more than three *Phaeoisaria* species are present, suggesting an extraordinarily high species diversity of this genus associated with *Magnolia delavayi* Franch.

To address the remaining taxonomic uncertainties in *Phaeoisaria* highlighted by the new species described in this study, and considering the existing research gaps in this genus, future research should focus on the following key aspects: (1) Expand taxon sampling in understudied habitats (e.g., fungal-associated niches, subtropical forests, high-altitude areas) and supplement strains from the clade in Fig. 1 to clarify taxonomic status and provide comprehensive sample support. (2) Construct multi-gene datasets (including *rpb2*, *tef1α* and other protein-coding markers) for all known *Phaeoisaria* species to improve phylogenetic resolution and resolve unstable clades and synonym controversies. (3) Develop genus-specific molecular barcodes and standardize *rpb2* and *tef1α* as complementary barcodes for rapid and accurate species identification, which will facilitate future taxonomic surveys. (4) Investigate sexual morphs, conidia dimorphism on cultures, trophic modes (e.g., mycoparasitism, symbiosis), and ecological functions of *Phaeoisaria* species to clarify their roles in nutrient cycling and environmental responses, thereby filling critical knowledge gaps in their biology and ecology.

## Supplementary Material

XML Treatment for Phaeoisaria bailongtanensis

XML Treatment for Phaeoisaria clematidis

XML Treatment for Phaeoisaria magnoliicola

XML Treatment for Phaeoisaria qujingensis

## Additional information

### Artificial Intelligence (AI) use

The authors accept full responsibility for the content of the manuscript, including the disclosure of any use of AI.

The following AI tools were used in the preparation of this manuscript:

**AI tools**: DeepSeek, Doubao.

**Used for**: Language, style and writing.

**Description**: AI language tools (DeepSeek and Doubao) were used solely for grammatical and stylistic polishing of the manuscript, to improve the clarity and readability of the English text. No AI tool was used to generate original content, interpret data, or draw scientific conclusions. All authors take full responsibility for the accuracy and integrity of the work.

### Author ORCIDs

Q.-J. Shang https://orcid.org/0000-0002-2861-3058

J. Yang https://orcid.org/0000-0001-8158-2919

K.D. Hyde https://orcid.org/0000-0002-2191-0762

N.N. Wijayawardene https://orcid.org/0000-0003-0522-5498

A.M. Elgorban https://orcid.org/0000-0003-3664-7853

F.Z. Filimban https://orcid.org/0000-0001-7527-1313

W. Watsuntorn https://orcid.org/0009-0002-3196-4753

D.-Q. Dai https://orcid.org/0000-0001-8935-8807

### Data availability

All of the data that support the findings of this study are available in the main text or Supplementary Information.

## Supplementary materials

Supplementary material 1 Phylogram generated from ML analyses for *Phaeoisaria*ITS gene Data type jpg https://binary.pensoft.net/file/1721586This dataset is made available under the Open Database License (http://opendatacommons.org/licenses/odbl/1.0/). The Open Database License (ODbL) is a license agreement intended to allow users to freely share, modify, and use this Dataset while maintaining this same freedom for others, provided that the original source and author(s) are credited. Qiu-Ju Shang, Jing Yang, Kevin D. Hyde, Nalin N. Wijayawardene, Hind A. AL-Shwaiman, Abdallah M. Elgorban, Faten Zubair Filimban, Wannapawn Watsuntorn, Dong-Qin Dai

Supplementary material 2 Phylogram generated from ML analyses for *Phaeoisaria*LSU gene Data type jpg https://binary.pensoft.net/file/1721587This dataset is made available under the Open Database License (http://opendatacommons.org/licenses/odbl/1.0/). The Open Database License (ODbL) is a license agreement intended to allow users to freely share, modify, and use this Dataset while maintaining this same freedom for others, provided that the original source and author(s) are credited. Qiu-Ju Shang, Jing Yang, Kevin D. Hyde, Nalin N. Wijayawardene, Hind A. AL-Shwaiman, Abdallah M. Elgorban, Faten Zubair Filimban, Wannapawn Watsuntorn, Dong-Qin Dai

Supplementary material 3 Phylogram generated from ML analyses for *Phaeoisaria*SSU gene Data type jpg https://binary.pensoft.net/file/1721588This dataset is made available under the Open Database License (http://opendatacommons.org/licenses/odbl/1.0/). The Open Database License (ODbL) is a license agreement intended to allow users to freely share, modify, and use this Dataset while maintaining this same freedom for others, provided that the original source and author(s) are credited. Qiu-Ju Shang, Jing Yang, Kevin D. Hyde, Nalin N. Wijayawardene, Hind A. AL-Shwaiman, Abdallah M. Elgorban, Faten Zubair Filimban, Wannapawn Watsuntorn, Dong-Qin Dai

Supplementary material 4 Phylogram generated from ML analyses for *Phaeoisariarpb2* gene Data type jpg https://binary.pensoft.net/file/1721589This dataset is made available under the Open Database License (http://opendatacommons.org/licenses/odbl/1.0/). The Open Database License (ODbL) is a license agreement intended to allow users to freely share, modify, and use this Dataset while maintaining this same freedom for others, provided that the original source and author(s) are credited. Qiu-Ju Shang, Jing Yang, Kevin D. Hyde, Nalin N. Wijayawardene, Hind A. AL-Shwaiman, Abdallah M. Elgorban, Faten Zubair Filimban, Wannapawn Watsuntorn, Dong-Qin Dai
